# Three-Dimensional Assessment of Collum Angle in Different Sagittal Malocclusions: A Cross-Sectional Study

**DOI:** 10.7759/cureus.70418

**Published:** 2024-09-29

**Authors:** Sumedha Sen, Ranjana Singh, Susmita Majumder, Nungshinaro Tzudir, Soi Chakraborty, Manish Sharma, Seema Gupta

**Affiliations:** 1 Department of Orthodontics, Mithila Minority Dental College and Hospital, Darbhanga, IND; 2 Department of Dentistry, Ganesh Shankar Vidyarthi Medical College, Kanpur, IND; 3 Department of Dentistry, Aditya Diagnostics and Hospital, Dibrugarh, IND; 4 Department of Dentistry, Jain Clinic, Dimapur, IND; 5 Department of Dentistry, Apollo Hospitals, Kolkata, IND; 6 Department of Oral Pathology, Jawahar Medical Foundations Annasaheb Chudaman Patil Dental College, Dhule, IND; 7 Department of Orthodontics, Kothiwal Dental College and Research Centre, Moradabad, IND

**Keywords:** angle, crown, malocclusion, root, sagittal

## Abstract

Introduction: Assessment of crown-root angulation of the anterior teeth as defined by the collum angle (CA) is crucial for diagnosis and treatment planning. This study was undertaken to evaluate the CA of all anterior teeth within both the maxillary and mandibular arches across various sagittal malocclusions using cone-beam computed tomography (CBCT). Additionally, the secondary aims included investigating sex disparities and side variances in this angular measurement.

Materials and methods: This cross-sectional study was conducted on 80 pre-orthodontic patients divided into four groups with 20 patients in each group: Group 1, dentoskeletal class I; Group 2, dentoskeletal class II with division 1 malocclusion; Group 3, dentoskeletal class II with division 2 malocclusion; and Group 4, dentoskeletal class III malocclusion. CA was assessed using CBCT for all anterior teeth in both jaws. The data were then subjected to statistical analysis.

Results: There were statistically significant differences in the CA for all anterior teeth in both jaws among all groups (p<0.05). The mean CA was highest in Group 3, followed by Groups 4, 2, and 1 for the maxillary central incisors (CIs). The mean CA was highest in Group 3, followed by Group 2, Group 1, and Group 3 for the maxillary lateral incisors (LI). The mean CA was highest in Group 3, followed by Groups 2, 4, and 1 for the maxillary canines. The mean CA was highest in Group 3, followed by Groups 4, 2, and 1 for the mandibular CI. The mean CA was highest in Group 3, followed by Groups 4, 2, and 1 for the mandibular LI. The mean CA was highest in Group 3, followed by Groups 4, 2, and 1 for the mandibular canines. There were no statistically significant sex or side (right and left) differences between the groups (p>0.05).

Conclusion: The highest CA was observed in class II division 2 for all the anterior teeth. The CA of the mandibular teeth was lower than that of the maxillary teeth, and the lowest values of CA were obtained for mandibular teeth in class I malocclusion.

## Introduction

Variability in dental morphology intrinsically influences occlusion and its associated three-dimensional spatial relationships. A primary variation in the anatomical structure is the axial inclination of the tooth. During the assessment of axial inclination, the analysis is typically restricted to the crown surface with the assumption that the root is in the same plane as the crown. However, when evaluating an anterior tooth, there may be a notable divergence between the longitudinal axes of the tooth’s crown and its root [1]. The angle formed between these two longitudinal axes is referred to as the crown-to-root angle, also known as the collum angle (CA), and is utilized to establish the angular discrepancy between the two axes [2]. It is very important to estimate this angle in the anterior teeth, particularly in the field of implantology, as the placement of an angled abutment leads to the development of stresses in the cervical area, in the field of esthetic dentistry, this angle will influence both the durability and esthetic or cosmetic efficacy of the rehabilitative procedure, and in the field of orthodontics, as the force needs to pass through the long axis of the tooth [3].

Issrani et al. observed that in class II division 2 malocclusion, this angle decreased, and males exhibited an increased angle compared to females [4]. However, they assessed the CA only for the maxillary central incisors (CI). In class II division 2, maxillary CI is retroclined with decreased CA, whereas lateral incisors (LI) are labially placed with increased CA. Piya et al. found decreased CA in the maxillary CI of class II division 2 and mandibular LI was associated with class III malocclusion. The CA increased in mandibular canines with class III malocclusion. Conversely, the CA was negative in the mandibular CI of class II division 2 [5].

Most studies have assessed the CA using lateral cephalograms; however, it is a two-dimensional radiograph with superimposition, and therefore cannot provide information about all the anterior teeth and compare the right and left sides [2, 6]. Currently, it is possible to visualize the anterior teeth in three dimensions using cone-beam computed tomography (CBCT); hence, right- and left-side comparisons are also possible [1].

Owing to the limited availability of CBCT research on this subject, this study was undertaken with the principal aim of evaluating the CA of all anterior teeth within both the maxillary and mandibular arches across various malocclusions. Additionally, the secondary aims included investigating sex disparities and side variances in this angular measurement.

## Materials and methods

Study design and settings

A cross-sectional, observational study was conducted on the patients who reported to the Department of Orthodontics, Kothiwal Dental College and Research Centre, from July 2022 to April 2024. Written informed consent was obtained from all the patients before starting the study, maintaining utmost confidentiality. The study adhered to the principles of the Declaration of Helsinki and followed the Strengthening the Reporting of Observational Studies in Epidemiology (STROBE) guidelines for observational studies. Approval was obtained from the ethical review board before the initiation of the study (KDCRC/IERB/06/2022/08).

Sample size estimation

The sample size calculation was performed using GPower software (version 3.0. The estimation focused on detecting differences in the CA related to the maxillary CI among various classes of molar malocclusions [3]. A minimum total sample size of 76 was deemed sufficient based on an alpha level of 0.05 (margin of error), statistical power of 95%, and effect size of 0.4 [3]. To allow for equal representation across the four groups (class I, class II division 1; class II division 2; and class III), the final sample size was increased to 80 (20 per group).

Patient’s eligibility criteria

Eighty patients were selected based on the following inclusion criteria: healthy pre-orthodontic patients who underwent CBCT examination for diagnostic reasons that were not related to this study, presence of all the anterior teeth in both the arches, periodontally healthy anterior teeth, normal frenal attachments, and presence of average growth pattern (mandibular plane angle between sella-nasion plane and mandibular lower border of 26-370). Individuals with a history of orthodontic treatment, periodontal surgery, esthetic or restorative treatment of the anterior teeth, presence of systemic disease, pregnant and lactating females, medications that can affect the periodontium, smokers, and presence of anterior teeth anomalies were excluded from the study.

Methodology

The maxilla-mandibular angle (ANB) was used to assess the skeletal pattern of the individual on pre-treatment lateral cephalograms of the patients, which were taken as an essential diagnostic aid for all orthodontic patients before starting their treatment. The ANB angle was constructed between point A (the deepest point in the anterior concavity of the maxilla), point N (the most prominent point on the frontonasal suture), and point B (the deepest point on the concavity of the anterior mandible). Eighty patients were divided into three groups with 20 patients in each group: Group 1, dentoskeletal class I (ANB angle of 0-40); Group 2, skeletal class II with dental class II division 1 (ANB > 40); Group 3, skeletal class II with dental class II division 2 (ANB > 40); and Group 4, skeletal class III with dental class III (ANB < 00). Dental malocclusions were divided according to Angle’s system of classification of malocclusion [7].

CA was traced on CBCT scans of the patients where CBCT was obtained at 90 kV, with a mean exposure time of 6.4 seconds at 4 milliamperes, voxel size of 0.2 mm, focal spot of 0.5 mm, radiation, and field of view measuring 8 × 10 cm. Image reconstruction for visual analysis was performed using CS imaging software (CS Imaging Software Version 8. Carestream Dental, Atlanta, Georgia). The associated CBCT Digital Imaging and Communications in Medicine (DICOM) files were anonymized by transforming all identifiable data into a randomized numerical representation. This numerical identifier was documented in an Excel spreadsheet, along with all other relevant patient information. The chosen records served as the basis for CA assessment in the anterior teeth.

**Figure 1:**
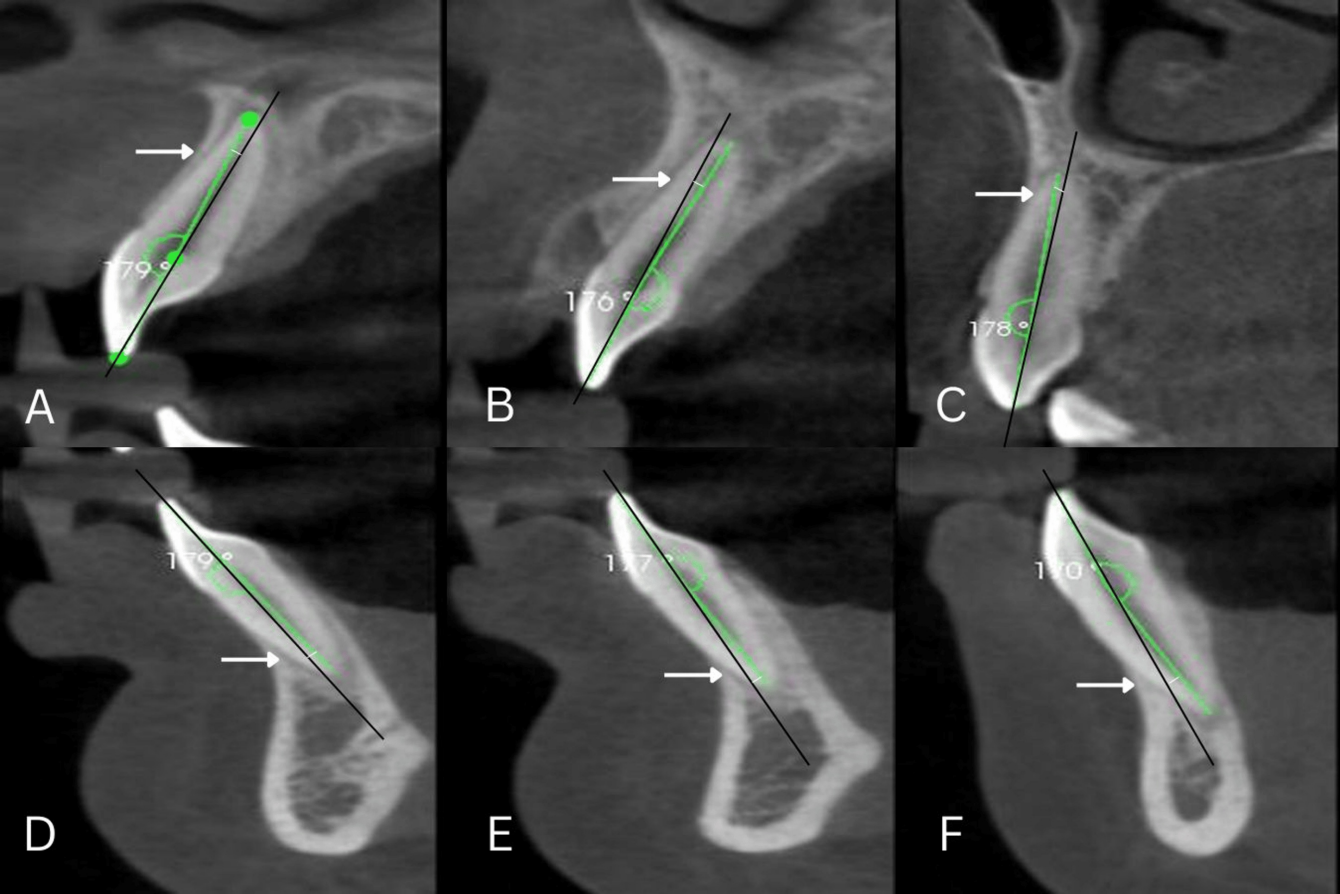
Collum angle (arrow mark) (A) maxillary central incisor, (B) maxillary lateral incisor, (C) maxillary canine, (D) mandibular central incisor, (E) mandibular lateral incisor, and (F) mandibular canine.

Three reference points were used to determine the CA angle (x). The first point was designated as the point on the intact incisal edge of the CI, LI, and the cusp tip of the canines. The second point was defined by a midpoint on the cementoenamel junction linking the facial and lingual surfaces, while the third point corresponded to the apex of the root. CA is the angle between the long axis of the crown (line passing from A to B) and the long axis of the root (line passing from B-C) [5]. The CA was calculated by deducting the angle (x) form by the two axes (A-B and B-C) from 180°. Consequently, the mathematical representation for the CA can be expressed as 180°minus x, and similar measurements were performed on the right and left sides, as well as for both the maxillary and mandibular anterior teeth (Figure 1).

Reliability

Prior to the commencement of the evaluation, 20 CBCT images and lateral cephalograms, which were excluded from the final sample, were employed for calibration purposes and underwent dual evaluation separated by a two-week interval. The intra-examiner concordance exhibited an exceptional degree of reliability (weighted kappa =0.94 for the CA and 0.89 for the ANB angle in the lateral cephalogram), thereby indicating that the examiners achieved a high level of calibration necessary for conducting the subjective assessment. A single observer (SM) traced all lateral cephalograms to assess the skeletal and growth patterns of individuals. Intra-observer reliability was assessed in randomly selected 20 individuals at one-week intervals by the same observer, who was blinded to the previous measurements. The intraclass correlation coefficient (ICC) was used for the evaluation and was found to be 97%. The CBCT measurements were performed by two observers (RS and SS) who were blinded to the group allocation. Intra- and inter-observer reliabilities were assessed in randomly selected 20 individuals at two-week intervals by the same observers who were blinded to the previous measurements. The ICC values showed 94% intra-observer reliability and 92% inter-observer reliability, indicating excellent reliability and reproducibility of the measurements. Paired-sample t-tests revealed no statistically significant discrepancies between the initial and subsequent measurements (P >0.05), indicating the absence of notable systematic errors. The statistician (MS) who performed the statistical analysis was blinded to group allocation.

Statistical analysis

Data analysis was conducted using the Statistical Package for Social Sciences (SPSS) version 21.0 (IBM Corp. Released 2013. IBM SPSS Statistics for Windows, Version 22.0. Armonk, NY: IBM Corp.). The CA values were reported as the mean and standard deviation. The normality of the data was assessed using the Shapiro-Wilk test, which confirmed that the data followed a normal distribution. As the data were continuous and normally distributed, parametric tests were employed for inferential statistics. One-way ANOVA and repeated-measures ANOVA were used to evaluate the results. Specifically, one-way ANOVA was used to assess the significance of class-wise malocclusion and racial differences in CA, whereas the independent t-test was used to examine sex-wise differences. Post-hoc pairwise comparisons were performed using the Bonferroni test. Statistical significance was set at P < 0.05.

## Results

The baseline characteristics of the sample revealed that the mean age of the patients in Group 1 was 26.50±3.61 years, Group 2 was 26.40±4.67 years, Group 3 was 28.18±3.77 years, and Group 4 was 25.50±3.20 years. The sample consisted of more males than females. Group 1 included 11 males (13.75%) and nine females (11.2%). Group 2 included eight males (10%) and 12 females (15%). Group 3 included 13 males (16.25%) and seven females (8.75%). Group 4 included 12 males (15%) and eight females (10%). This showed that males had more malocclusions than females, and females had more class II division 1 malocclusions (Table 1).

**Table 1:** Basic characteristics of groups. Data is presented in the form of mean ± standard deviation (SD), and n (%).

Sagittal pattern	Age (years)	Gender n (%)	
	Mean±SD	Male 44 (55.00)	Female 36 (45.00)
Group 1	26.50 ± 3.61	11 (13.75)	9 (11.25)
Group 2	26.40 ± 4.67	8 (10.00)	12 (15.00)
Group 3	28.18 ± 3.77	13 (16.25)	7 (8.75)
Group 4	25.50 ± 3.20	12 (15.00)	8 (10.00)

**Figure 2:**
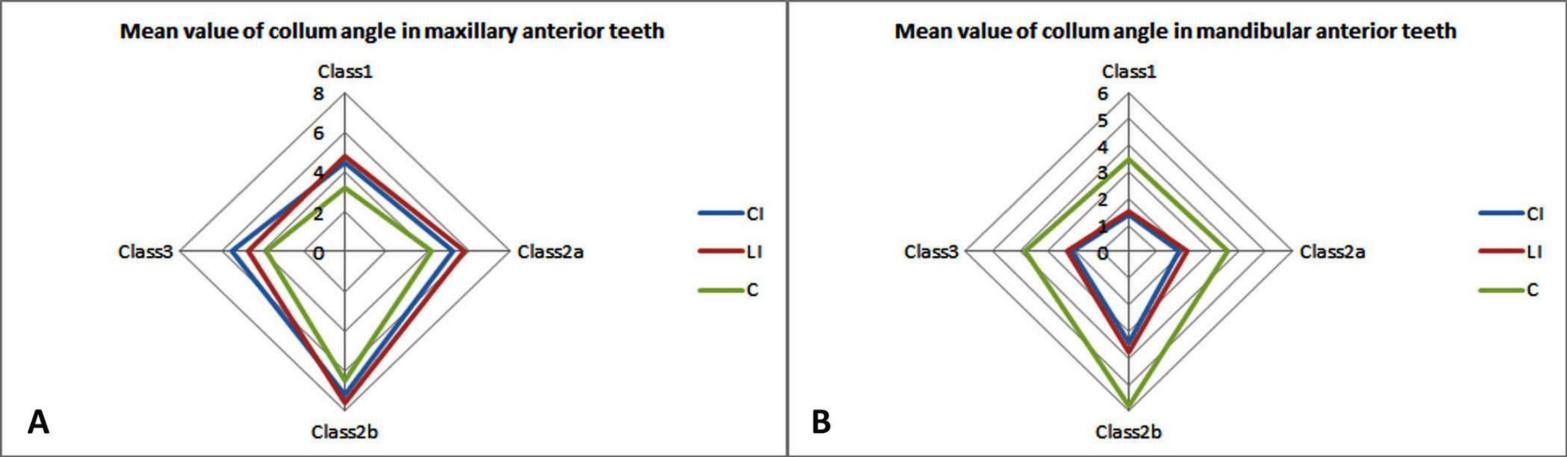
Mean collum angle (CA) values in different groups for (A) maxillary anterior teeth and (B) mandibular anterior teeth. CI: central incisor; LI: lateral incisor; C: canine

Because there were no statistically significant differences between the right and left sides, the values of the right and left sides were averaged for further analysis. There was a statistically significant difference in the mean CA between the groups for all teeth in both jaws (Figure 2).

The mean CA was highest in Group 3, followed by Groups 4, 2, and 1 for the maxillary CI. Post-hoc analysis further revealed that the values were statistically different between Groups 1 and 3 and between Groups 2 and 3. The mean CA was highest in Group 3, followed by Group 2, Group 1, and Group 3 for the maxillary LI. Post-hoc analysis further revealed that the values were statistically different between Groups 1 and 3. The mean CA was highest in Group 3, followed by Groups 2, 4, and 1 for the maxillary canines. Post-hoc analysis further revealed that the values were statistically different between Groups 1 and 3, Groups 2 and 3, and Groups 3 and 4 (Tables 2-3).

**Table 2:** Comparison of mean values of collum angle (CA) between groups by ANOVA test. *P <0.05: significant

Jaw	Sagittal plane	N	Central incisor	p value	Lateral incisor	p value	Canine	p value
			Mean±SD		Mean±SD		Mean±SD	
Maxilla	Group 1	20	4.5±1.3	0.003*	4.8±1.4	0.002*	3.2±1.5	0.001*
	Group 2	20	5.2±1.8		5.8±1.9		4.2±1.6	
	Group 3	20	7.2±2.6		7.6±3.2		6.5±2.3	
	Group 4	20	5.5±1.7		4.7±1.8		3.8±1.2	
Mandible	Group 1	20	1.4±0.56	0.001*	1.5±0.67	0.001*	3.5±1.3	0.009*
	Group 2	20	1.8±0.23		2.1±0.54		3.6±1.6	
	Group 3	20	3.4±1.3		3.8±1.7		5.8±2.9	
	Group 4	20	2.1±0.98		2.3±1.2		3.8±1.7	

**Table 3:** Pairwise comparison with Bonferroni post-hoc test. *P <0.051: significant

Tooth	Pairwise comparison		Maxilla		Mandible	
			Mean at 95% CI	p value	Mean at 95% CI	p value
Central incisor	Group 1	Group 2	-0.8859 to 2.2859	0.650	-0.3214 to 1.1214	0.469
	Group 1	Group 3	1.1141 to 4.2859	0.002*	1.2786 to 2.7214	0.001*
	Group 1	Group 4	-0.5859 to 2.5859	0.350	-0.0214 to 1.4214	0.061
	Group 2	Group 3	0.4141 to 3.5859	0.007*	0.8786 to 2.3214	0.001*
	Group 2	Group 4	-1.2859 to 1.8859	0.950	-0.4214 to 1.0214	0.695
	Group 3	Group 4	-3.2859 to -0.1141	0.030*	-2.0214 to -0.5786	0.001*
Lateral incisor	Group 1	Group 2	-0.8128 to 2.8128	0.470	-0.3352 to 1.5352	0.339
	Group 1	Group 3	0.9872 to 4.6128,	0.007*	1.3648 to 3.2352	0.001*
	Group 1	Group 4	-1.9128 to 1.7128	0.990	-0.1352 to 1.7352	0.120
	Group 2	Group 3	-0.0128 to 3.6128	0.520	0.7648 to 2.6352	0.001*
	Group 2	Group 4	-2.9128 to 0.7128	0.380	-0.7352 to 1.1352	0.943
	Group 3	Group 4	-4.7128 to -1.0872	0.004*	-2.4352 to -0.5648	0.000*
Canine	Group 1	Group 2	-0.4109 to 2.4109	0.250	-1.5378 to 1.7378	0.999
	Group 1	Group 3	1.8891 to 4.7109	0.001*	0.6622 to 3.9378	0.002*
	Group 1	Group 4	-0.8109 to 2.0109	0.680	-1.3378 to 1.9378	0.963
	Group 2	Group 3	0.8891 to 3.7109,	0.003*	0.5622 to 3.8378	0.004*
	Group 2	Group 4	-1.8109 to 1.0109	0.870	-1.4378 to 1.8378	0.989
	Group 3	Group 4	-4.1109 to -1.2891	0.001*	-3.6378 to -0.3622	0.010*

The mean CA was highest in Group 3, followed by Groups 4, 2, and 1 for the mandibular CI. Post-hoc analysis further revealed that the values were statistically different in Groups 1 vs. 3, 2 vs. 3, and 3 vs. 4. The mean CA was highest in Group 3, followed by Groups 4, 2, and 1 for the mandibular LI. Post-hoc analysis further revealed that the values were statistically different between Groups 1 and 3 and between Groups 3 and 4. The mean CA was highest in Group 3, followed by Groups 4, 2, and 1 for the mandibular canines. Post-hoc analysis further revealed that the values were statistically different between Groups 1 and 3, Groups 2 and 3, and Groups 3 and 4 (Tables 2-3).

No statistically significant differences were noted between the sexes for any of the teeth in both the maxillary and mandibular jaws (p > 0.05). Although the mean CA values were not significant, females had higher values than males (Table 4).

**Table 4:** Gender-wise comparison of groups by Independent t-test. M: male; F: female; data presented in the form of mean ± standard deviation (SD).

Jaw	Groups	Gender	N	Central incisor	p value	Lateral incisor	p value	Canine	p value
				Mean±SD		Mean±SD		Mean±SD	
Maxilla	Group 1	M	11	4.35±1.18	0.621	4.73±0.96	0.805	3.15±1.02	0.743
		F	9	4.52±1.21		4.81±1.08		3.26±1.08	
	Group 2	M	8	4.96±1.21	0.426	5.65±1.21	0.655	4.18±1.25	0.862
		F	12	5.25±1.26		5.82±1.18		4.25±1.28	
	Group 3	M	13	7.16±3.12	0.908	7.25±3.14	0.649	5.92±2.16	0.364
		F	7	7.28±3.18		7.71±3.21		6.56±2.25	
	Group 4	M	12	5.13±1.56	0.322	4.31±1.23	0.308	3.68±1.23	0.621
		F	8	5.65±1.72		4.72±1.28		3.87±1.18	
Mandible	Group 1	M	11	1.34±0.56	0.707	1.45±0.45	0.423	1.29±0.67	0.760
		F	9	1.41±0.61		1.58±0.56		1.35±0.56	
	Group 2	M	8	1.75±0.55	0.713	2.12±1.09	0.933	1.54±0.72	0.614
		F	12	1.82±0.63		2.15±1.16		1.67±0.89	
	Group 3	M	13	3.23±1.23	0.603	3.72±1.24	0.792	2.85±1.09	0.725
		F	7	3.45±1.42		3.83±1.38		2.98±1.23	
	Group 4	M	12	2.06±0.89	0.532	2.21±0.76	0.610	1.56±0.92	0.531
		F	8	2.23±0.96		2.34±0.84		1.75±0.98	

## Discussion

This study was conducted to evaluate CA in different dentoskeletal malocclusions in the anterior teeth. The results of this study indicated that the CA significantly differed between the different malocclusion groups. No statistically significant differences were observed between the right and left sides. This finding is in agreement with previous studies by Elangovan et al. [1] and Issrani et al. [4]. However, Issrani et al. did not evaluate differences between the right and left sides.

The mean CA was highest in class II division 2 for all maxillary anterior teeth, whereas it was lower in class I and class II division 1 malocclusion for maxillary CI, class I and class III for maxillary LI, and canines. According to Feres et al. [6] and Shen et al. [2], the mean CA was the highest for class II division 2 for maxillary CI. This phenomenon may be attributed to the heightened pressure exerted by the lower lip in class II division 2, which obscures the maxillary CI by approximately 5 mm, resulting in a modification of the crown-root angle. It is posited that there could be potential bending of the tooth in the cervical region due to the pressure exerted by the lower lip during the developmental and eruptive phases of the incisors [8]. Class II division 2 shows predominantly retroclined maxillary CI, which has a high genetic component [9].

CA was highest for maxillary LI and mandibular canines, which is in accordance with a study by Elangovan et al. [1]. This could be due to the fact that the mandibular canines endure considerable functional forces attributable to their essential role in the processes of mastication, specifically in the actions of tearing and facilitating lateral movements during occlusion. The elevation of the CA may contribute to a more efficient distribution of these forces between the dental crown and the root structure. The maxillary LI is instrumental in influencing the esthetic appearance of the smile, and its angulation aids in its alignment with the dental arch and contours of the lips. An increased CA permits the crown to exhibit an outward curvature, while simultaneously ensuring that the root remains securely anchored within the surrounding osseous tissue. In both instances, elevated CA facilitates the adaptation of the teeth to the particular functional and esthetic requirements imposed upon them [10].

CA was highest for all mandibular teeth in class II division 2, lowest for class III CI, and class I LI and canines. In class II division 2 malocclusion, maxillary incisors are generally positioned in a retroclined manner. This retroclination influences the angulation of mandibular incisors, particularly as a reaction to modified occlusal dynamics. CA is often elevated in all mandibular teeth, as these teeth may adjust for the retroclined position of the maxillary incisors. Conversely, in class III malocclusion, owing to the presence of mandibular prognathism, the lower CI tends to exhibit a more upright or even retroclined posture to mitigate the effects of the anterior crossbite. In class I occlusion, the mandibular LI and canines typically demonstrate a more neutral or normative alignment. Given the absence of marked dental compensation or skeletal discrepancies as observed in class II or class III malocclusions, the CA is usually lower, indicating a more harmonious relationship between the crown and root structures [1,10,11].

CA plays a pivotal role in the expression of torque when employing a straight-wire appliance because it is assumed that the morphology of the facial surfaces of all anterior teeth remains consistent across patients. Furthermore, previous studies have illustrated that the application of an identical archwire in conjunction with preadjusted brackets yielded disparate torque expressions owing to discrepancies in labial crown morphology and angulation between the crown and root [11-13]. Pai et al. demonstrated that in individuals exhibiting elevated CA, the rotational center shifted cervically, resulting in an increased stress-strain distribution, while concurrently diminishing the degree of intrusion [14].

The results of this study did not report any significant sex differences in CA between the groups, although females showed higher CA values than males. Issrani et al. [4] reported significant sex differences in the mean CA of the maxillary CI, with males showing higher values than females. The disparity in the findings could be explained by the fact that Issrani et al. [4] assessed CA for maxillary CI, whereas in this study, CA was evaluated for all anterior teeth. The diminished jaw size in females may lead to an increased compensatory angulation of the dentition, including the CA, to accommodate the reduced dimensions of the dental arch and uphold optimal occlusion [15]. Females frequently display a more retroclined positioning of the anterior teeth than males. This phenomenon may stem from disparities in soft tissue profiles and dental esthetics between the sexes [16]. To adapt to these variations, CA may be more pronounced in females to assist in preserving both functional and esthetic integrity. Conversely, males tend to undergo a prolonged phase of mandibular growth, culminating in a larger and more protruded mandible. This growth pattern can influence the overall alignment and angulation of the teeth, potentially resulting in diminished CA in males [17].

Clinical implications

Class II division 2 patients have increased CA. Acknowledging this anatomical characteristic is essential when devising strategies to procline or align the anterior teeth, ensuring that there are no detrimental impacts on the roots or surrounding periodontal tissues. Teeth with increased CA are at higher risk of root resorption during orthodontic treatment, particularly if heavy forces are applied, as their roots are more buccally placed toward the labial cortical plate.

Limitations

The major limitation of this study was the lack of evaluation of the CA in different vertical malocclusions. The ANB angle was used to assess the skeletal relationship, which failed to identify the problem in a specific jaw. The occurrence of artifacts in CBCT imaging is another limitation of this investigation. Despite taking all the necessary precautions to minimize the artifacts, a limited number of images were affected by noise, which obstructed clear visualization of the apex of the upper CI root.

## Conclusions

Assessing CA across various sagittal malocclusions concerning the anterior teeth has significant clinical relevance in orthodontic diagnosis, treatment strategy formulation, and resultant efficacy. The results of this study showed that the highest CA was observed in class II division 2 for all the anterior teeth. The CA of the mandibular teeth was lower than that of the maxillary teeth, and the lowest values of CA were obtained for mandibular teeth in class I malocclusion. The highest CA was observed in the maxillary LI and the mandibular canines. No statistically significant differences were noted between the sexes for all the teeth in both the maxillary and mandibular jaws.
